# Artificial Intelligence‐assisted Endoscopy and Examiner Confidence: A Study on Human–Artificial Intelligence Interaction in Barrett's Esophagus (With Video)

**DOI:** 10.1002/deo2.70150

**Published:** 2025-06-19

**Authors:** David Roser, Michael Meinikheim, Anna Muzalyova, Robert Mendel, Christoph Palm, Andreas Probst, Sandra Nagl, Markus W. Scheppach, Christoph Römmele, Elisabeth Schnoy, Nasim Parsa, Michael F. Byrne, Helmut Messmann, Alanna Ebigbo

**Affiliations:** ^1^ Department of Gastroenterology University Hospital Augsburg Augsburg Germany; ^2^ Regensburg Medical Image Computing (ReMIC), Ostbayerische Technische Hochschule Regensburg Regensburg Germany; ^3^ Division of Gastroenterology and Hepatology Mayo Clinic Scottsdale USA; ^4^ Satisfai Health Vancouver Canada; ^5^ Division of Gastroenterology, Vancouver General Hospital Vancouver Canada; ^6^ Department of Medicine I St. Josef‐Hospital Bochum Germany

**Keywords:** AI, artificial intelligence, Barrett's esophagus, endoscopy, HAII

## Abstract

**Objective:**

Despite high stand‐alone performance, studies demonstrate that artificial intelligence (AI)‐supported endoscopic diagnostics often fall short in clinical applications due to human‐AI interaction factors. This video‐based trial on Barrett's esophagus aimed to investigate how examiner behavior, their levels of confidence, and system usability influence the diagnostic outcomes of AI‐assisted endoscopy.

**Methods:**

The present analysis employed data from a multicenter randomized controlled tandem video trial involving 22 endoscopists with varying degrees of expertise. Participants were tasked with evaluating a set of 96 endoscopic videos of Barrett's esophagus in two distinct rounds, with and without AI assistance. Diagnostic confidence levels were recorded, and decision changes were categorized according to the AI prediction. Additional surveys assessed user experience and system usability ratings.

**Results:**

AI assistance significantly increased examiner confidence levels (*p* < 0.001) and accuracy. Withdrawing AI assistance decreased confidence (*p* < 0.001), but not accuracy. Experts consistently reported higher confidence than non‐experts (*p* < 0.001), regardless of performance. Despite improved confidence, correct AI guidance was disregarded in 16% of all cases, and 9% of initially correct diagnoses were changed to incorrect ones. Overreliance on AI, algorithm aversion, and uncertainty in AI predictions were identified as key factors influencing outcomes. The System Usability Scale questionnaire scores indicated good to excellent usability, with non‐experts scoring 73.5 and experts 85.6.

**Conclusions:**

Our findings highlight the pivotal function of examiner behavior in AI‐assisted endoscopy. To fully realize the benefits of AI, implementing explainable AI, improving user interfaces, and providing targeted training are essential. Addressing these factors could enhance diagnostic accuracy and confidence in clinical practice.

## 1 Introduction

In recent years, the integration of artificial intelligence (AI) into medical practice has brought about transformative changes in various medical fields [1, 2, 3, 4], with endoscopy being no exception [5]. Specifically, the potential application of AI in the evaluation of Barrett's esophagus (BE) holds immense promise in improving diagnostic accuracy [6, 7, 8, 9], by analyzing endoscopic images in real‐time [10] and aiding in early detection. BE‐related neoplasia (BERN) poses a particularly challenging condition for novice and expert endoscopists alike. Traditional endoscopic evaluation, while effective if carried out by expert endoscopists, is inherently subjective and prone to interobserver variability. This underscores the necessity for more objective and reliable diagnostic tools.

A critical aspect that has emerged in the discussion surrounding the integration of AI in endoscopy is human‐AI interaction (HAII). Research has demonstrated that endoscopists, even with the utilization of AI systems, do not attain the stand‐alone performance level of AI [11, 12, 13, 14, 15, 16]. The reasons for this shortcoming are still poorly understood, but they are essential for optimizing the integration of AI into endoscopic practice and maximizing its clinical utility in the future.

In this analysis, we sought to understand why human‐AI collaborations fail to achieve the stand‐alone performance of AI algorithms when operated in isolation. The analysis evaluated the effects of overreliance and complacency, algorithm aversion, algorithm uncertainty, and system usability on the diagnostic performance and confidence of endoscopists.

## 2 Methods

### 2.1 Study Design and Participants

**FIGURE 1:**
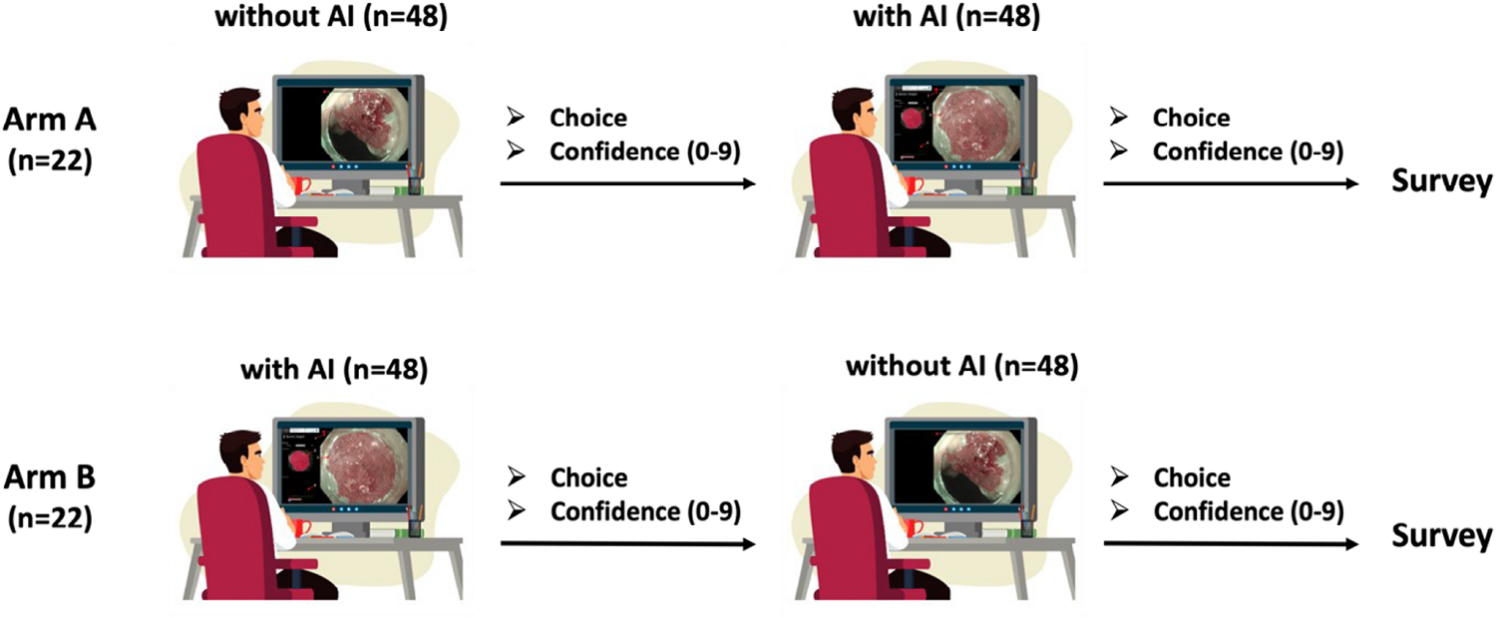
Study design. Examiners assessed the 96 standardized endoscopic videos of Barrett's Esophagus in two separate arms, without artificial intelligence (AI) assistance first, followed by a review with AI assistance (Arm A), and vice versa in Arm B.

This analysis utilized data from a previously conducted multicenter randomized controlled tandem video trial [8], which examined the impact of the underlying AI system (*Verit‐AI*) on the performance of endoscopists in the assessment of BE. In the current analysis, we focused on evaluating the confidence levels of expert and non‐expert examiners, depending on the correctness of AI predictions. In the preceding study, 22 endoscopists from 12 centers and four different countries with varying levels of experience in BE evaluation were included. Participants were divided into an expert (*n* = 4) and non‐expert group (*n* = 18), on the basis of years of experience in Barrett's assessment and expertise in the treatment of BERN. Endoscopic videos (*n* = 96) were block randomized into two blocks utilizing non‐dysplastic BE (NDBE) and BERN using a custom R script. Examiners were then tasked to assess each video block (*n* = 48) in two rounds of a different order: first without AI and subsequently, with AI support—defined as Arm A and vice versa—defined as Arm B. The study design is shown in Figure 1. We analyzed the recorded confidence levels on a scale from 0 to 9 after each assessment. Examiners were permitted to re‐watch the video and amend their prediction during one round at will. After the second round, participants were asked to fill out a Likert scale survey designed to identify factors potentially influencing their confidence levels, including experience, familiarity with AI, and perceived accuracy of AI and the System Usability Scale questionnaire (SUS; Data S1). Each item in the SUS is scored on a scale from 0 to 4, resulting in a grading between excellent (score 85 or above), good (70–84), acceptable (50–69), and poor usability (below 50). Finally, we assessed the mode of output presentation (i.e., the stability of the AI output during lesion presentation) and linked this to the diagnostic confidence and performance of the participating endoscopists.

### 2.2 Video Dataset

The video dataset comprised 96 endoscopic videos from 72 patients evaluated for BE and BERN at the University Hospital of Augsburg. The dataset comprised overview and close‐up videos of varying lengths (15 s to 1 min and 30 s) to simulate real‐life conditions. The endoscopic videos were selected to represent all stages of Barrett's cascade, with a high proportion of NDBE (*n* = 45) according to the higher general prevalence. To adequately evaluate the assessment of dysplasia, the BERN lesions were divided into LGD (*n* = 5), HGD (*n* = 7), T1a (*n* = 36), and T1b (*n* = 3) tumors. Videos were also selected based on the feasibility of external evaluation and incorporation of virtual chromoendoscopy (VCE) or near‐focus mode. Videos with insufficient visibility or less than 30 s were excluded from the study. All cases had histological confirmation by specialized pathologists.

### 2.3 Data Analysis

We analyzed the changes in diagnostic confidence levels between the two rounds (without and with AI). The present study examined the associations between confidence levels and the accuracy of AI predictions, stratifying by correct and incorrect AI advice. AI predictions were categorized into the following groups, depending on the defined ground truth of the lesion: “True positive” (TP), “False positive” (FP), “True negative” (TN), and “False negative” (FN). The positive predictions were further categorized into stable (SP) and non‐stable (NSP) predictions, depending on the duration of the AI output visible on the user interface (Data S2 and S3). SP was defined as a segmentation heatmap displayed for more than 3 s (150 consecutive frames). NSP implied cases where the segmentation map repeatedly appeared at a consistent location for a cumulative duration exceeding 3 s (150 frames) but not continuously [8].

### 2.4 Statistical Methods

Changes in confidence levels were analyzed using the Wilcoxon signed‐rank test for dependent samples and the Mann‐Whitney‐U test for comparisons between independent groups. The choice of non‐parametric tests was based on non‐normal data distribution. A two‐sided significance level of <0.05 was employed for all statistical analyses. If not stated otherwise, results are presented as mean ± standard deviation (SD). For statistical analyses, *SPSS* version 28.0 and *Microsoft Excel* version 16.86 were utilized.

## 3 Results

### 3.1 Confidence Levels With and Without AI

**FIGURE 2:**
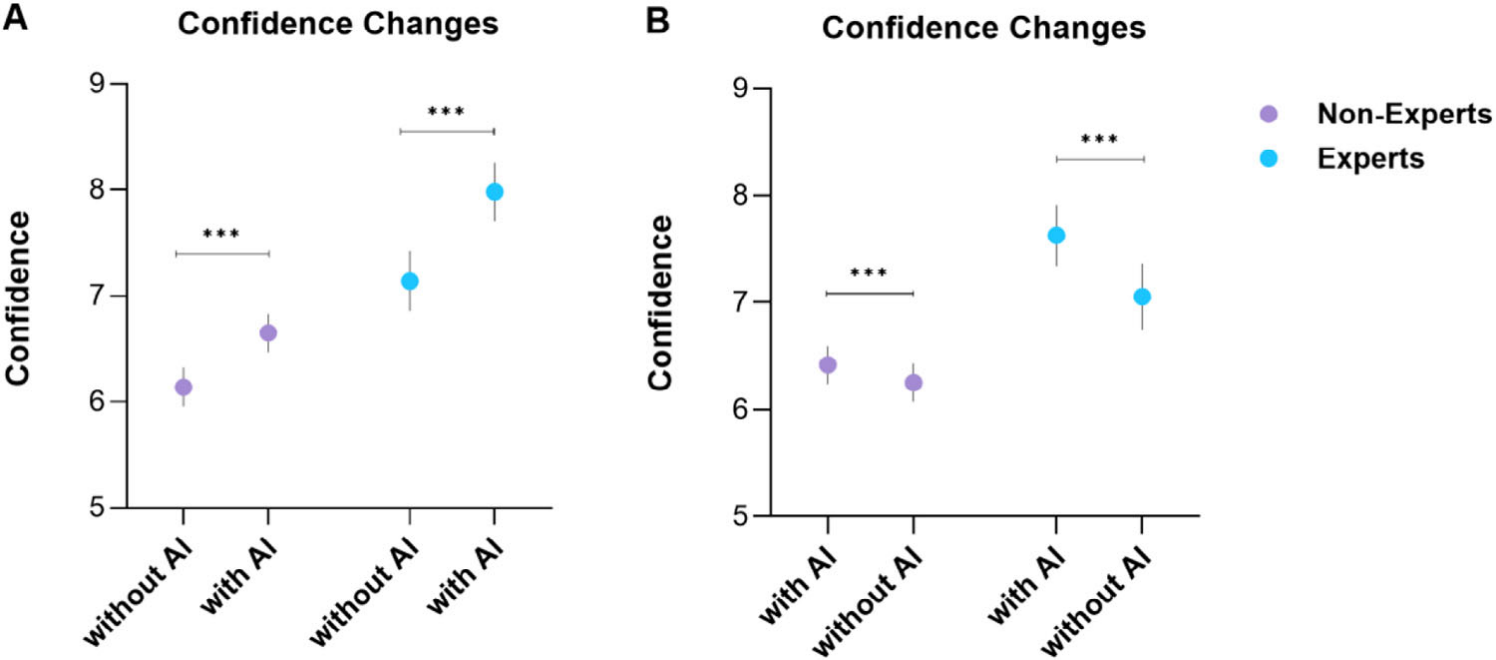
Confidence changes without and with artificial intelligence (AI) in Arm A (A) and B (B), (*p* < 0.001 ***, mean with 95% confidence interval).

The integration of AI (Arm A) significantly boosted examiner confidence levels across both expert and non‐expert groups (*p* < 0.001), as demonstrated in Figure 2. Experts consistently demonstrated higher levels of confidence in comparison to non‐experts, regardless of AI presence (*p* < 0.001). Notably, when AI support was withdrawn (Arm B), a significant reduction in confidence was observed (*p* < 0.001).

### 3.2 Decision Changes

#### 3.2.1 Frequency and Accuracy of Cecision Changes

AI support led to a notable increase in the frequency of decision changes among participants. Of these changes, 75.2% resulted in a transition from incorrect to correct outcome. Confidence levels in cases where participants corrected their initial incorrect predictions rose significantly (*p* = 0.028). The stability of AI recommendations was an associated factor, with SP accounting for 82.2% of successful corrections. Conversely, in instances of erroneous corrections, only 37.5% SP were observed. Overall, the utilization of AI‐driven recommendations resulted in significantly more successful than wrongful corrections (*p* < 0.001).

#### 3.2.2 Impact on Confidence

Changes from incorrect to correct diagnoses were associated with an average confidence increase from 3.44 to 4.23 (*p* = 0.028). Conversely, changes from correct to incorrect diagnoses, while less frequent, showed no statistically significant confidence difference, as shown in Table 1.

**TABLE 1:** Confidence levels before and after decision changes, stratified by participant group and direction of change.

		Confidence		
Decision change	Group	without AI	with AI	*p*‐value
**False to true** *n* = 91	All	3.44	4.23	**0.028**
	Non‐expert	3.37	4.07	0.073
	Expert	3.77	4.94	0.152
**True to false** *n* = 31	All	2.80	3.77	0.10
	Non‐expert	2.65	3.10	0.483
	Expert	3.10	5.10	0.095

### 3.3 Disregarding AI Recommendations

In 16% of cases, the examiners disregarded correct AI suggestions, resulting in incorrect diagnoses. Subgroup analysis revealed that experts were significantly more likely than non‐experts to change a correct initial decision to an incorrect one with the correct AI suggestion (*p* = 0.042). In contrast, non‐experts exhibited a significantly higher probability of transitioning from an incorrect to a correct decision when provided with a correct AI suggestion (*p* = 0.042). The absolute and relative proportions of *decision types* and *decision changes* for each scenario are shown in Tables 2 and 3.

**TABLE 2:** Analysis of overall decisions relative to all predictions (%) divided by subgroup, most probable contributing factor, and potential action point.

Potential action point	Bias/factor	Decision type (with AI)	Non‐experts	Experts	Overall
**Correct outcome**					
–	**Examiner experience**	**True to true** (with correct AI)	43.62% (*n* = 451)	17.21% (*n* = 178)	**60.83%** (*n* = 629)
		**True to true** (with incorrect AI)	3.58% (*n* = 37)	1.26% (*n* = 13)	**4.84%** (*n* = 50)
–	**Benefit of AI**	**False to true** (with correct AI)	6.77% (*n* = 70)	1.55% (*n* = 16)	**8.32%** (*n* = 86)
	**(Benefit of AI)**	**False to true** (with incorrect AI)	0.39% (*n* = 4)	0.10% (*n* = 1)	**0.48%** (*n* = 5)
**Incorrect outcome**					
Targeted training	**Algorithm aversion**	**True to false** (with correct AI)	0.58% (*n* = 6)	0.48% (*n* = 5)	**1.06%** (*n* = 11)
		**False to false** (with correct AI)	10.93% (*n* = 113)	3.97% (*n* = 41)	**14.89%** (*n* = 154)
	**Overreliance**	**True to false** (with incorrect AI)	1.45% (*n* = 15)	0.48% (*n* = 5)	**1.93%** (*n* = 20)
Improving AI performance	**Lack of AI accuracy**	**False to false** (with incorrect AI)	5.42% (*n* = 56)	2.22% (*n* = 23)	**7.64%** (*n* = 79)

**TABLE 3:** Analysis of decision changes relative to all changes made (%) divided by subgroup and most probable contributing factor.

Bias/factor	Decision change (with AI)	Non‐experts	Experts	Overall
Benefit of AI	**False to true** (with correct AI)	57.38% (*n* = 70)	13.11% (*n* = 16)	**70.49%** (*n* = 86)
(Benefit of AI)	**False to true** (with incorrect AI)	3.28% (*n* = 4)	0.82% (*n* = 1)	**4.10%** (*n* = 5)
Algorithm aversion	**True to false** (with correct AI)	4.92% (*n* = 6)	4.10% (*n* = 5)	**9.02%** (*n* = 11)
Overreliance	**True to false** (with incorrect AI)	12.30% (*n* = 15)	4.10% (*n* = 5)	**16.39%** (*n* = 20)

### 3.4 Case‐Level Error Analysis and Difficult Videos

To identify the most common sources of error, we conducted a case‐level analysis of all videos. “Difficult” cases were defined as those in which more than 50% of examiners made incorrect classifications. Out of the 96 videos evaluated, 10 met this criterion (six NDBE and four BERN). Most FP occurred in NDBE cases with surface irregularities and within Barrett's “tongues” or islands, which may have mimicked dysplasia. In BERN cases, frequent errors were linked to heatmap instability or NSP, long‐segment BE, or inconspicuous features leading to localization mismatches.

### 3.5 System Usability Score

The SUS revealed notable differences in perceived usability between non‐experts and experts. Non‐experts recorded a mean score of 73.54 ± 12.77, classified as “good” usability, whereas experts assigned a markedly higher mean score of 85.63 ± 13.75, categorized as “excellent.”

### 3.6 Survey and User Experience

#### 3.6.1 User Characteristics

Non‐experts reported an average of 10 years of clinical practice, during which they assessed approximately 60 BE cases annually, leading to a cumulative total of 19 treated BE cases over their careers. Conversely, experts had an average of 15 years of experience, with an annual caseload of 71 BE cases and a total of 33 treated cases.

#### 3.6.2 Survey Results

Of the 22 participants, 16 completed the survey, including all experts and 12 non‐experts. The survey evaluated multiple dimensions of user experience through a 5‐point Likert scale, where 1 represented “never” or “very difficult,” and 5 indicated “always” or “very easy.” Participants generally reported limited prior exposure to AI‐assisted endoscopy, with an average score of 2.06 ± 1.0. The video trial and the clinical cases were perceived as moderately challenging, with mean scores of 3.06 ± 0.68 and 2.69 ± 0.6, respectively. Despite the perceived complexity, the AI interface was deemed minimally intrusive, receiving a low disruption score of 1.87 ± 0.89. Additionally, AI predictions were rated positively for their trustworthiness in both NDBE and BERN cases, with mean scores of 3.81 ± 0.54 and 3.44 ± 0.81, respectively.

**FIGURE 3:**
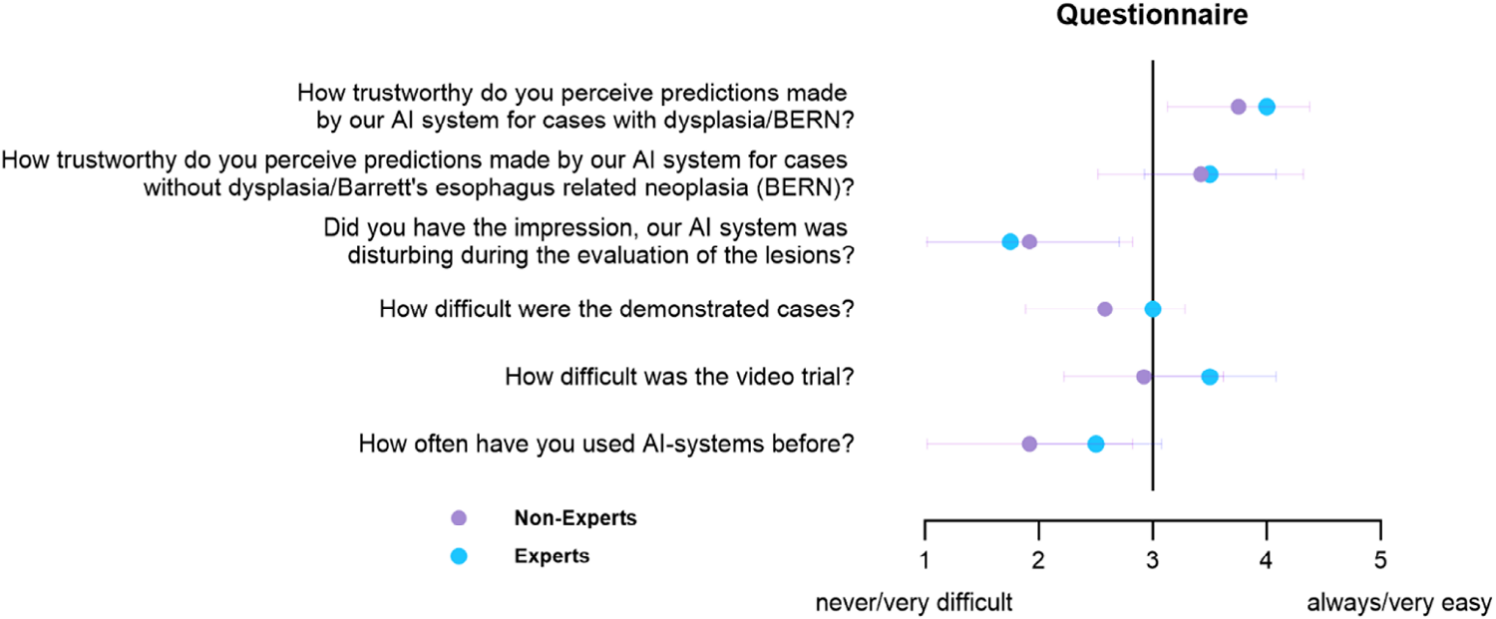
Survey results illustrating user experience ratings stratified by participant expertise.

No statistically significant differences were observed between the two groups regarding their overall user experience, as shown in Figure 3.

### 3.7 Recommendability

Participants were also queried about the recommendability of AI systems in different clinical environments. The results showed that 87.5% endorsed AI use for inexperienced examiners and 81.3% for experienced examiners in hospitals. 75% supported AI use for experienced examiners in private practices, and only 56.3% for Barrett's experts.

## 4 Discussion

**FIGURE 4:**
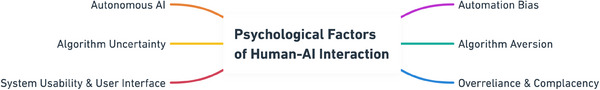
Psychological factors influencing human‐artificial intelligence interaction.

As AI systems continue to improve—potentially achieving sensitivities and specificities unattainable even by highly specialized experts—the necessity of scrutinizing AI decisions might need reconsideration. Our study seeks to understand why—as evidenced by numerous other studies [11, 13–17] ‐ AI systems do not live up to their experimental performance in clinical real‐life settings, or proxies like our video trial. While the use of AI did increase accuracy [8] and confidence in this setting and led to a significant increase in correct decision changes, examiners or endoscopists frequently made incorrect decisions despite adequate AI guidance (15.95% of decisions disregarded correct AI guidance). Withdrawing the AI overlay, however, did not decrease accuracy but confidence. As recently summarized [18, 19, 20], we need to account for numerous possible pitfalls and cognitive biases in the interaction between endoscopists/human examiners and the mechanical AI system, as shown in Figure 4.

### 4.1 Discrepancy Between Human Performance With AI and the Stand‐alone Performance of AI

One of the most notable findings of this study is that, despite the increase in the number of correct choices made with AI guidance (an 8.3% increase in correct decision changes, which was significantly higher in non‐experts), correct AI indications were still disregarded in nearly 16% of all cases displayed. Moreover, 9% of the decision changes were from an initially correct choice to an incorrect one, despite correct AI indications. These results are consistent with previous studies describing this discrepancy [8, 11, 12, 13, 14, 15, 16] and highlight critical issues in the integration of AI into clinical practice.

### 4.2 Algorithm Aversion

Especially experienced examiners showed reluctance to trust AI recommendations, as evidenced by the significantly higher change rate to incorrect decisions. This behavior, possibly explained by algorithm aversion, may stem from a preference for personal expertise over AI input [21]. Our data further showed that experts maintained higher confidence than non‐experts, regardless of AI accuracy. This higher confidence may have influenced their decision‐making and overreliance on personal judgment rather than AI recommendations. To reduce algorithm aversion and the “black box” issue of deep learning algorithms, developing explainable AI (XAI) systems that clarify their decision‐making processes or offer real‐time confidence scores could improve trust and adherence to AI suggestions [22, 23, 24].

### 4.3 Algorithm Uncertainty

Cases where examiners incorrectly changed their decisions due to wrong AI guidance were associated with lower confidence levels. When correlated with the stability of the assessment, it was evident that the AI also faced difficulties, displaying a high proportion (45.8%) of NSP, suggesting “alignment of uncertainty” [25].

Improving interaction might involve the AI providing a probabilistic prediction, thereby expressing its own uncertainty. Although our study did not specifically test probabilistic outputs, future research could explore whether presenting AI confidence levels influences examiner adherence and decision‐making. AI systems should aim to include a quality indicator, possibly including movement speed and inspection time, in order to provide sufficient suggestions. Trust in AI could also be enhanced by clarifying the database it was trained on (‘model cards’) [26], or by providing pre‐use instructional modules or case‐based tutorials.

### 4.4 Overreliance, Complacency, and Automation Bias

Another aspect hindering optimal AI use is an overreliance on AI‐generated predictions, thereby diminishing their critical evaluation of diagnostic probability [27, 28, 29]. This was noticeable in the 16.4% of incorrect *decision changes* according to respective incorrect AI indications. This phenomenon applies equally to both FP‐ and FN outcomes. Furthermore, the data obtained revealed no statistically significant differences between experts and non‐experts in this regard. The use of AI within this context could potentially prevent novices from refining their assessment techniques, thus limiting their performance in unassisted examinations (known as “deskilling”) [27, 30]. Notably, there is a lack of longitudinal data examining the long‐term impact of AI on endoscopic training and performance.

Moreover, there is a tendency for examiners to override their own clinical judgment due to overconfidence in the system's capabilities (complacency), as described in the setting of AI‐assisted mammography or histopathological assessment [31, 32]. Despite this, AI assistance increased examiner confidence levels, but this did not always consistently translate into improved diagnostic accuracy, suggesting that automation bias [27, 28] may have enhanced confidence without necessarily enhancing outcomes.

**FIGURE 5:**
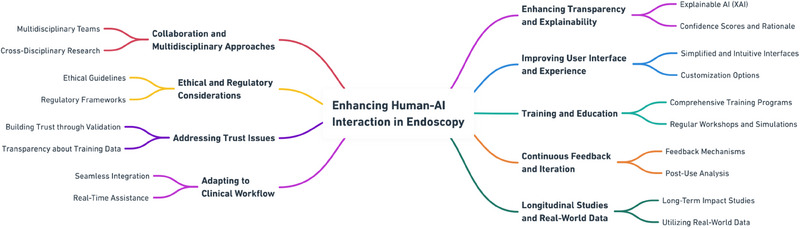
The authors' recommendations for improvement of human‐artificial intelligence interaction in artificial intelligence in medicine.

Prospective clinical trials, however, indicate that “blind trust” in AI systems could potentially enhance overall examiner performance, for example in differentiating hyperplastic polyps from adenomas in the distal colon [33]. When translated to our data, around 16% more cases would be assessed correctly. This is in line with the high stand‐alone diagnostic accuracy demonstrated by current AI technologies. To maximize the benefits of AI‐assisted endoscopy, targeted training is essential. We propose a modular approach combining basic AI literacy (e.g., understanding algorithmic limitations and output), exposure to common failure scenarios through annotated case reviews, and simulation‐based training with real‐time feedback [34], as summarized in Figure 5.

### 4.5 System Usability and User Interface

The SUS scores indicated good to excellent usability, with no objectively significant reasons for these shortcomings. Moreover, the survey revealed that the acceptance and credibility of AI among examiners were satisfactorily high. The only notable criticism was the partially incomprehensible interface (e.g., traffic lights and bars). In subsequent individual interviews, examiners often could not pinpoint specific reasons for disregarding the AI, citing that it “did not feel right.” Occasionally, the presentation of lesions did not match the personal examination style of the endoscopist (e.g., dwell time on the lesion or magnification level). We still recommend designing the interface to be more user‐friendly and intuitive. Overall, the AI system was primarily recommended for less experienced examiners.

### 4.6 Limitations of the Study

Due to current healthcare regulations in Germany, conducting a real‐world clinical trial of the AI system used in this study was not possible. Consequently, we employed a video‐based, randomized trial design, which has inherent limitations. These include the passive nature of video review, the inability to account for individual examination techniques or duration, and the lack of real‐time use of advanced imaging technologies (VCE) or acetowhitening. To partially mitigate these limitations, narrow‐band imaging and texture and color enhancement imaging sequences were included where applicable, and an adequate dwell time was provided during the video review process. The same set of videos was utilized in both study arms without a washout phase, which may have contributed to higher confidence levels in Arm B, indicating a potential training effect. However, this approach ensured consistency and uniform difficulty across both arms. To further elucidate the relationship between unstable AI predictions and examiner uncertainty, a larger sample size would be required. Moreover, in real‐life settings, technical issues such as slow processor speeds, lagging, frozen screens, or “alarm fatigue” can create an aversion to using the software and increase the cognitive burden on endoscopists [35]. These effects, however, were not tested for in this controlled video‐trial setting.

Ultimately, we are convinced that AI will permanently integrate into medicine, particularly in endoscopy. However, we should not only focus on ever‐improving sensitivities and larger datasets but also consider the impact of AI on the human factor and vice versa. We need to explore options to enhance our collaboration with our new colleague, AI.

## Ethics statement

The study was conducted following the ethical standards of the institutional and national research committees and the Helsinki Declaration. Approval was obtained from the ethics committee of the Ludwig‐Maximilians‐University of Munich (PNO: 20‐010).

## Consent

Informed consent was secured from all participants.

## Conflicts of Interest

Robert Mendel declares support from the Bavarian Institute for Digital Transformation and BayWiss Gesundheit. Robert Mendel has received consulting fees from Satisfai Health. Sandra Nagl has received lecture fees from Microtech, Falk Pharma, Sanofi, and Pfizer. Michael F. Byrne has stock options from Satisfai Health. Markus W. Scheppach has received consulting fees from Olympus Germany. Nasim Parsa has received consulting fees from Phathom Pharmaceuticals and CapsoVision and lecture fees from the American College of Gastroenterology. NP has stock options from Satisfai Health. All other authors declare no conflicts of interest.

## Clinical trial registration

n/a

## Supporting information




**DATA S1** Post‐intervention survey.

**DATA S2** Video examples of Stable Prediction in BERN and NDBE.
**VIDEO S1** Examples of video cases presented to the examiners with a stable prediction of the AI overlay. Stable predictions were defined as a segmentation heat map displayed for more than 3 s (150 consecutive frames).

**DATA S3** Video examples of Nonstable Prediction in BERN and NDBE.
**VIDEO S2** Examples of video cases presented to the examiners with a non‐stable prediction of the AI overlay. Non‐stable prediction implied cases where the segmentation map repeatedly appeared at the same spot for an overall cumulative time of more than 3 s (150 frames) but not continuously.

## Data Availability

The datasets used and/or analyzed during the current study are available from the corresponding author upon reasonable request.
