# Supplementary material for: Artificial Intelligence‐assisted Endoscopy and Examiner Confidence: A Study on Human–Artificial Intelligence Interaction in Barrett's Esophagus (With Video)
Source: DEN Open. 2025 Jun 19;6(1):e70150. doi: 10.1002/deo2.70150 (PMC12178752; doi:10.1002/deo2.70150)
Supplement: Supplementary file 1 — DATA S1 Post‐intervention survey. [file DEO2-6-e70150-s003.docx]

**Survey & System Usability Scale**

1. Baseline questions:

How many years of experience do you have in the field of endoscopy?

____________________[years]

How many Barrett´s cases do you see on average per year?

_____________________[cases/year]

How many Barrett´s cases do you treat (ESD, Ablation, EMR) per year?

_____________________[cases/year]

How many Barrett´s cases did you already treat (ESD, Ablation, EMR)?

_____________________[N]

1. User Experience
2. How often have you used AI-systems (any) before?
3. – Never 2 – Rarely 3 – Sometimes 4 – Often 5 – Always
4. How difficult was the video-trial?

1 – Very difficult 2 – Difficult 3 – Neutral 4 – Easy 5 – Very easy

1. How difficult were the demonstrated cases?

1 – Very difficult 2 – Difficult 3 – Neutral 4 – Easy 5 – Very easy

1. Did you have the impression, our AIS was disturbing during the evaluation of the lesions?

1 – Never 2 – Rarely 3 – Sometimes 4 – Often 5 – Always

1. How trustworthy do you perceive predictions made by our AIS for cases **without** dysplasia/Barrett´s esophagus-related neoplasia (BERN)?

1 – Never 2 – Rarely 3 – Sometimes 4 – Often 5 – Always

1. How trustworthy do you perceive predictions made by our AIS for cases **with** dysplasia/BERN?

1 – Never 2 – Rarely 3 – Sometimes 4 – Often 5 – Always

1. Would you recommend using AI as a clinical decision support system in clinical practice?
   1. No
   2. Yes, for following experience levels:

*(multiple choice possible)*

1- endoscopist (but no specialist) 0-5 years of experience

2- endoscopist (specialist/ gastroenterologist) >5 years of experience (working in a hospital)

3- endoscopist (specialist/ gastroenterologist) >5 years of experience (working in private practice)

4- expert (Barrett-expert)

1. In case you can´t recommend using this or any similar AI-system in clinical practice, please elaborate on your motives.

__________________________________________________________________________________________________________________________________

_________________________________________________________________

1. Usability

Please answer the following questions to describe the user-experience during the test (System Usability Scale)

1. I think that I would like to use this AI-system frequently

Strongly disagree Strongly agree

|  |  |  |  |  |
| --- | --- | --- | --- | --- |
| 1 | 2 | 3 | 4 | 5 |

1. I found the AI-system unnecessarily complex

Strongly disagree Strongly agree

|  |  |  |  |  |
| --- | --- | --- | --- | --- |
| 1 | 2 | 3 | 4 | 5 |

1. I thought the AI-system was easy to use

Strongly disagree Strongly agree

|  |  |  |  |  |
| --- | --- | --- | --- | --- |
| 1 | 2 | 3 | 4 | 5 |

1. I think that I would need the support of a technical person to be able to use this AI-system

Strongly disagree Strongly agree

|  |  |  |  |  |
| --- | --- | --- | --- | --- |
| 1 | 2 | 3 | 4 | 5 |

1. I found the various functions in this AI-system were well integrated

Strongly disagree Strongly agree

|  |  |  |  |  |
| --- | --- | --- | --- | --- |
| 1 | 2 | 3 | 4 | 5 |

1. I thought there was too much inconsistency in this AI-system

Strongly disagree Strongly agree

|  |  |  |  |  |
| --- | --- | --- | --- | --- |
| 1 | 2 | 3 | 4 | 5 |

1. I would imagine that most people would learn to use this AI-system very quickly

Strongly disagree Strongly agree

|  |  |  |  |  |
| --- | --- | --- | --- | --- |
| 1 | 2 | 3 | 4 | 5 |

1. I found the AI-system very cumbersome to use

Strongly disagree Strongly agree

|  |  |  |  |  |
| --- | --- | --- | --- | --- |
| 1 | 2 | 3 | 4 | 5 |

1. I felt very confident using the AI-system

Strongly disagree Strongly agree

|  |  |  |  |  |
| --- | --- | --- | --- | --- |
| 1 | 2 | 3 | 4 | 5 |

10. I needed to learn a lot of things before I could get going with this AI-system

Strongly disagree Strongly agree

|  |  |  |  |  |
| --- | --- | --- | --- | --- |
| 1 | 2 | 3 | 4 | 5 |

Open questions

What would you change in the interface of our AIs to make it more comprehensible/intuitive?

_________________________________________________________________________________________________________________________________________________________________________________________________________
